# Dysregulated RNA m6A Methylation Contributes to the Metastasis of Gallbladder Cancer Through miR‐146a‐5p

**DOI:** 10.1111/jcmm.70764

**Published:** 2025-08-10

**Authors:** Yuhui Liu, Ruizhi He, Wenjia Wang, Qilong Xia, Di Zhang, Shutao Pan, Min Wang, Qi Zhang, Simiao Xu, Jun Gong, Renyi Qin

**Affiliations:** ^1^ Department of Biliary‐Pancreatic Surgery, Affiliated Tongji Hospital, Tongji Medical College Huazhong University of Science and Technology Wuhan Hubei China; ^2^ Hubei Key Laboratory of Hepato‐Pancreato‐Biliary Diseases, Affiliated Tongji Hospital, Tongji Medical College Huazhong University of Science and Technology Wuhan Hubei China; ^3^ Division of Child Healthcare, Department of Pediatrics, Affiliated Tongji Hospital, Tongji Medical College Huazhong University of Science and Technology Wuhan Hubei China; ^4^ Department of Plastic and Cosmetic Surgery, Affiliated Tongji Hospital, Tongji Medical College Huazhong University of Science and Technology Wuhan Hubei China; ^5^ Xianning Medical College Hubei University of Science and Technology Xianning Hubei China; ^6^ Department of Endocrinology, Affiliated Tongji Hospital, Tongji Medical College Huazhong University of Science and Technology Wuhan Hubei China

**Keywords:** gallbladder cancer (GBC), METTL14, METTL3, microRNA processing, miR‐146a‐5p, N6‐methyladenosine (m6A)

## Abstract

Gallbladder cancer (GBC) remains a challenging malignancy with a poor prognosis largely due to its highly metastatic nature and lack of effective treatment options. In this study, we investigated the role of N6‐methyladenosine (m6A) modification and its regulatory factors, METTL3 and METTL14, in GBC. Our results showed that m6A levels as well as the expression of METTL3 and METTL14 were significantly downregulated in GBC tissues compared to normal gallbladder tissues. In vitro experiments showed that manipulation of METTL3 and METTL14 expression regulated GBC cell migration and invasive ability, and the liver metastasis model of nude mice further demonstrated the involvement of m6A modification in regulating GBC metastasis. Further investigation identified miR‐146a‐5p as a downstream target regulated by m6A, with tumour‐suppressive effects on GBC cell migration and invasion. Overall, our findings provide new insights into the role of m6A modification and its regulation on microRNA in GBC pathogenesis and offer a potential strategy for the treatment of GBC.

## 1 Introduction

Gallbladder cancer (GBC) is a vicious malignancy of primary biliary tract [1]. Despite notable strides in the diagnosis and treatment of digestive malignancies, GBC remains a formidable adversary due to its high metastatic propensity and the absence of early symptoms [2]. Individuals diagnosed with GBC face a median survival time of less than 1 year, with a disheartening 5‐year survival rate below 10% [3, 4]. The mechanisms underlying the tumourigenesis and metastasis of GBC remain elusive. Consequently, further investigations are imperative to elucidate the underlying molecular mechanisms of GBC.

N6‐methyladenosine (m6A), initially identified in 1974, represents the most prevalent RNA modification in mammalian cells [5]. M6A methylation manifests as a dynamic and reversible process, orchestrated by a cadre of regulating genes, including writers (METTL3, METTL14, and WTAP), erasers (FTO and ALKBH5), and readers (YTH domain family and heterogeneous nuclear ribonucleoprotein (HNRNP) family) [6]. Recent years have seen advancements in m6A site mapping, revealing its predilection for the RRm6ACH (*R* = A/G, H = A, C or U) sequence motif, which serves as a preferred target of the METTL3/METTL14 complex [7]. During m6A installation, METTL3 operates as the catalytic subunit, while METTL14 aids in RNA binding [8]. M6A modification exerts a pivotal influence over mRNA stability, splicing, translation, and microRNA processing [9]. Studies have underscored m6A dysregulation, particularly aberrations in METTL3 and METTL14 levels, across various cancers, including breast, colorectal, liver, and lung cancers [10, 11]. However, the expression levels and functions of m6A, METTL3, and METTL14 in GBC remain elusive.

MicroRNAs (miRNAs), small non‐coding RNAs typically comprising a few nucleotides in length, serve as regulators of gene expression [12]. miRNAs usually lead to the degradation or translational inhibition of their target mRNAs by binding to their 3′ untranslated region (3′UTR) [13]. The biogenesis of miRNAs is initiated by RNA polymerase II to transcriptionally generate primary miRNAs (pri‐miRNAs). The pri‐miRNA is then cut by ribonuclease drosha and DGCR8 to form precursor miRNAs (pre‐miRNAs), followed by transport to the cytoplasm, where it is processed by dicer to form mature miRNAs [14]. Dysregulation of miRNAs contributes to numerous diseases, including cancer, and a plethora of miRNAs have been identified to be involved in the initiation and progression of GBC [15, 16].

In the present study, the decrement of the m6A level together with the downregulation of METTL3 and METTL14 in GBC tissues was confirmed. Furthermore, we demonstrated that METTL3 and METTL14 function as tumour suppressors by facilitating the maturation of miR‐146a‐5p, which in turn inhibited the migration and invasion abilities of GBC cells both in vitro and in vivo. These findings shed light on the role and function of m6A modification in GBC and offer a novel therapeutic strategy for GBC treatment.

## 2 Materials and Methods

### 2.1 Patients and Samples

GBC tissues along with adjacent normal gallbladder tissues were acquired from 12 patients who underwent surgery in Tongji Hospital (Wuhan, China). Informed consent for the use of specimens was obtained from all patients. The GBC tissue microarray and associated clinical data were obtained from Shanghai OUTDO BIOTECH CO. LTD. The study was conducted in accordance with the Declaration of Helsinki and approved by the Human Research Ethics Committee of Tongji Hospital of Huazhong University of Science and Technology.

### 2.2 Cell Lines and Reagents

The human gallbladder cancer cell lines GBC‐SD and NOZ were generously provided by Prof. Yingbin Liu, Xinhua Hospital, Shanghai Jiao Tong University School of Medicine, China. Cells were cultured in RPMI 1640 (Cytiva, MA, USA) supplemented with 10% fetal bovine serum in a humidified incubator at 37°C with 5% CO2. The following antibodies and reagents were purchased: METTL3 (A8370), METTL14 (A8530) from Abclonal (Wuhan, China); E‐cadherin (20874–1‐AP), N‐cadherin (22018–1‐AP), Vimentin (10366–1‐AP), DGCR8 (60084–1‐Ig), GAPDH (60004–1‐Ig), and β‐actin (81115–1‐RR) from Proteintech (Wuhan, China). Secondary antibodies used in this study were from Jackson ImmunoResearch (PA, USA).

### 2.3 RNA Extraction and Tissue m6A Content Quantification

Total RNA was extracted from the gallbladder tumour tissue and paired paratumor tissue from patients using a RNA‐easy isolation reagent (Vazyme, Nanjing, China). M6A levels in total RNA were measured using the EpiQuik m6A RNA methylation quantification kit (Epigentek Group Inc.) according to the manufacturer's instructions.

### 2.4 Protein Isolation and Western Blot Analysis

Cells or tissues were lysed with RIPA buffer supplemented with a protease inhibitor and subjected to sonication and centrifugation; supernatant was then collected, and protein concentration was measured using BCA methods. The western blot was carried out as previously reported [17].

### 2.5 Histology and Immunohistochemistry

Gallbladder tumours, along with corresponding para‐tumour tissue samples from patients, were paraffin‐embedded and subjected to immunohistochemistry (IHC) evaluation. H‐score was then calculated as previously reported [18]. For TMA analysis, METTL3 and METTL14 expression in a human GBC TMA was assessed and scored by two independent researchers according to the staining intensity and the percentage of positive cells.

### 2.6 Transwell Assay

Transwell assay was carried out by using 8‐μm pore size filters in a 24‐well plate (Corning, NY, USA). For the invasion assay, the filters were coated with Matrigel first, and 700 μL FBS‐containing medium was added to the lower chambers, and 3 × 10^4^ cells in 200 μL of medium without FBS were added to the upper chambers. After 24 h of incubation, cells in the upper chamber were removed using a cotton swab. The remaining cells were fixed and stained. Photographs were taken under a light microscope (Olympus, Tokyo, Japan), and the migrating cells were counted in three randomly selected fields in three replicate wells. The migration assay was conducted in a similar procedure only without coating the filter with Matrigel.

### 2.7 Wound Healing Assay

For the wound healing assay, 4 × 10^5^/well GBC‐SD or NOZ cells were seeded in a 6‐well plate and reached confluence. A wound was made using a 200 μL tip. Cells were then washed with PBS three times and incubated with serum‐free RPMI‐1640 for 24 h. Photographs were taken, and the distance between two sides of the wound was measured in three replicate wells.

### 2.8 Plasmid Construction, Transfection, and Lentivirus Production

Plasmids expressing Flag‐METTL3 and Flag‐METTL14 were generated using PCR and cloned into the pHAGE vector (Addgene, MA, USA). Short hairpin sequences targeting METTL3 and METTL14 were cloned into the pLKO.1 vector (Addgene). MiR‐146a‐5p agomir and control were purchased from Sangon biotech (Shanghai, China). The sequence of human primary miR‐146a was synthesised by Sangon biotech and cloned into the pCDH vector. M6A site point mutations were introduced by PCR‐based transformation, converting GGAC to GGCC. Lipofectamine 3000 (Invitrogen) was used for plasmid transfection. Sequences of primers used in the study are listed in Table S1. Lentivirus was produced using HEK293T cells as previously reported [18].

### 2.9 miRNA Sequencing

Total RNA was extracted from GBC‐SD cells transfected with shRNA targeting METTL3 or METTL14 as mentioned above. Next, RNA was sent to BGI for sequencing (Shenzhen, China). Briefly, small RNAs between 18 to 30 nucleotides were isolated by gel electrophoresis. After reverse transcription and PCR amplification, the miRNA libraries were assessed for quality using the Agilent 2100 Bioanalyzer and subjected to high‐throughput sequencing using the BGISEQ‐500 sequencer. Low‐quality reads were removed, and clean reads were aligned to the human genome (hg38) and miRbase. Raw counts were normalised, and R package DESeq2 was used to analyse the differently expressed miRNAs with the criteria of |log2 FC| > 1 and FDR < 0.05.

### 2.10 Quantitative Real‐Time PCR

After cell collection using an RNA Isolater, mRNA and miRNA were separately isolated using specific columns from the MiPure miRNA Kit (Vazyme). mRNA was reverse‐transcribed into cDNA using HiScript Q RT SuperMix (Vazyme), and miRNA was converted to cDNA using the miRNA 1st strand cDNA synthesis kit (by stem‐loop) (Vazyme). qRT‐PCR was performed using ChamQ Universal SYBR qPCR master mix for mRNA and miRNA unimodal SYBR qPCR master mix (Vazyme) for miRNA. GAPDH and U6 were used as internal controls for mRNA and miRNA, respectively. For the m6A site‐mutated constructs, puromycin expression was assessed concurrently to account for any variations in transfection efficiency. Primer sequences are provided in Table S1.

### 2.11 Analysis of GEO Datasets

GEO databases were searched for datasets including differentially expressed miRNA in GBC and normal gallbladder tissues. The miRNA profiling of 40 gallbladder tumour tissues and 8 non‐tumour gallbladder tissues from GSE104165 (Goeppert et al., 2019) was selected for further analysis. The microarray probes were reannotated to miRNAs, and expression levels of miRNAs in tumour and non‐tumour gallbladder tissues were extracted for comparison. R package sva was used to remove batch effect, and limma was used to analyse the differentially expressed miRNAs with the criteria of |log2 FC| > 2 and FDR < 0.05. Visualisation was carried out with ggplot2 and pheatmap.

### 2.12 Immunoprecipitation Assay

GBC‐SD cells transfected with Flag‐METTL3, Flag‐METTL14, or empty vector were harvested on ice and lysed with IP buffer (Servicebio) containing protease inhibitor. After sonication and centrifugation, the supernatant was collected and treated with or without RNase A (20 μg/mL, Yeasen Biotech, Shanghai, China), followed by incubation at 4°C for 6 h with Protein A/G magnetic beads (Bimake, TX, USA) and Anti‐METTL3, Anti‐METTL14, or Anti‐IgG primary antibody, respectively. Beads were then washed three times, resuspended with 2× SDS‐PAGE loading buffer, and subjected to western blot analysis.

### 2.13 Animal Experiments

Animal studies were approved by the Laboratory Animal Welfare and Ethics Committee of Tongji Hospital, Tongji Medical College, Huazhong University of Science and Technology. Female nude BALB/c mice were obtained from Vital River Laboratories (Beijing, China) and were divided into 9 groups. Each group included six independent biological replicates (*n* = 6). Stably transfected NOZ cells (2 × 10^6^ cells per mouse) were mixed with Matrigel at a ratio of 1:1 and were injected into the spleen of the mice. For the agomiR treatment group, cells were incubated with indicated agomiR for 48 h at a concentration of 200 nM prior to injection. After 4 weeks, all mice were sacrificed and livers were collected and photographed, fixed with 4% paraformaldehyde for HE staining. Metastatic nodules on the surface of the liver were counted and analysed.

### 2.14 Statistical Analysis

Data are presented as means ± SD from at least three independent experiments. *p* values were evaluated by Student's two‐tailed *t*‐test using GraphPad Prism 9 software (GraphPad Software Inc., CA, USA). *p* < 0.05 were considered statistically significant.

## 3 Results

### 3.1 Reduced m6a Modification and Decreased Expression of METTL3 and METTL14 in GBC Tissues Correlate With Metastasis and Poor Prognosis

**FIGURE 1:**
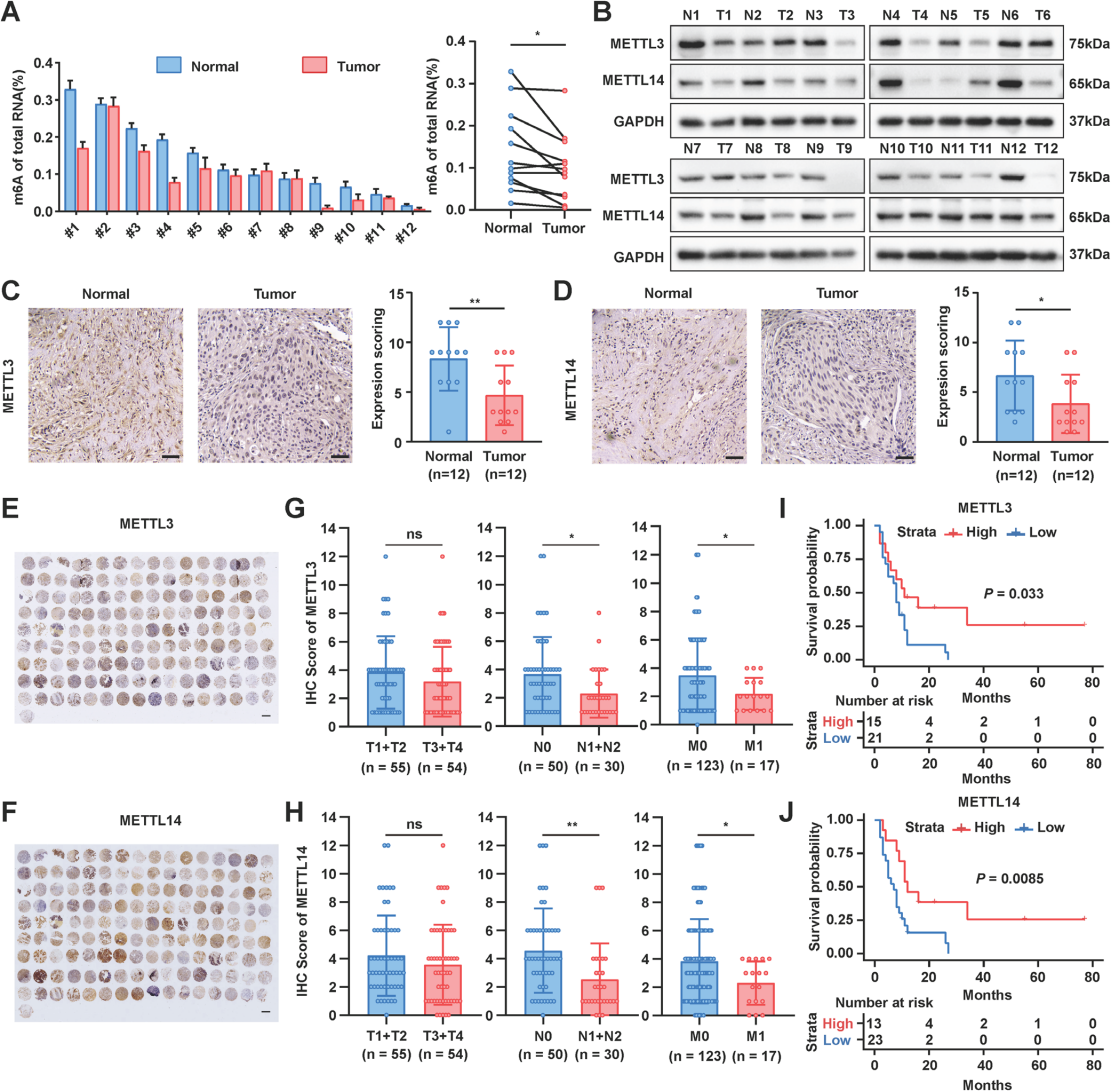
Downregulated m^6^A levels in GBC. (A) Quantification of m^6^A levels in 12 pairs of GBC and normal gallbladder tissues. (B) Western blot analysis of METTL3 and METTL14 expression in the same tissue pairs. (C, D) Immunohistochemistry analysis showing the expression levels of METTL3 and METTL14 in GBC and paired normal gallbladder tissues. Scale bar: 50 μm. (E, F) Images of the GBC tissue microarray showing the expression of METTL3 (E) or METTL14 (F). (G, H) Correlation between METTL3 (G) or METTL14 (H) expression and the TNM tumour stage of GBC patients. (I, J) Kaplan–Meier analysis assessing the correlation between METTL3 (I) or METTL14 (J) expression levels and patient survival. *p* values were calculated by Student's *t*‐test. *p < 0.05, **p < 0.01, ns not significant.

The alteration of m6A modification has been documented in various human tumours [19]. To investigate the status of m6A modification in GBC, we initially quantified m6A levels in human GBC tissues and pair‐matched normal tissues using the colorimetric m6A quantification methods. Our analysis revealed a decrease in m6A levels in GBC tissues compared to pair‐matched normal tissues in 9 out of 12 (75%) patients (Figure 1A). To elucidate the reason for the diminished m6A levels in GBC, we evaluated the expression of key m6A regulatory factors (METTL3 and METTL14) in paired cancer and normal tissue samples. Western blot analysis demonstrated significantly lower expression levels of METTL3 and METTL14 in GBC tissues than in paired normal tissues (Figure 1B). Immunohistochemistry staining further corroborated these findings, showing reduced average expression levels of METTL3 and METTL14 in GBC tissues compared with normal tissues (Figure 1C,D). We also examined the correlation between METTL3 and METTL14 expression and clinical data using a GBC tissue microarray. The results indicated that lower expression levels of METTL3 and METTL14 were associated with increased lymph node metastasis (N1‐N2), enhanced distal metastasis (M1), and poorer patient survival (Figure 1E–J). These results suggest that both m6A modification levels and the expression of METTL3 and METTL14 were reduced in GBC patients and that decreased METTL3 and METTL14 expression is linked to the lymph node and distal organ metastasis as well as worse patient survival outcomes.

### 3.2 Reduced m6A Modification Promoted the Invasive Capacity of GBC

**FIGURE 2:**
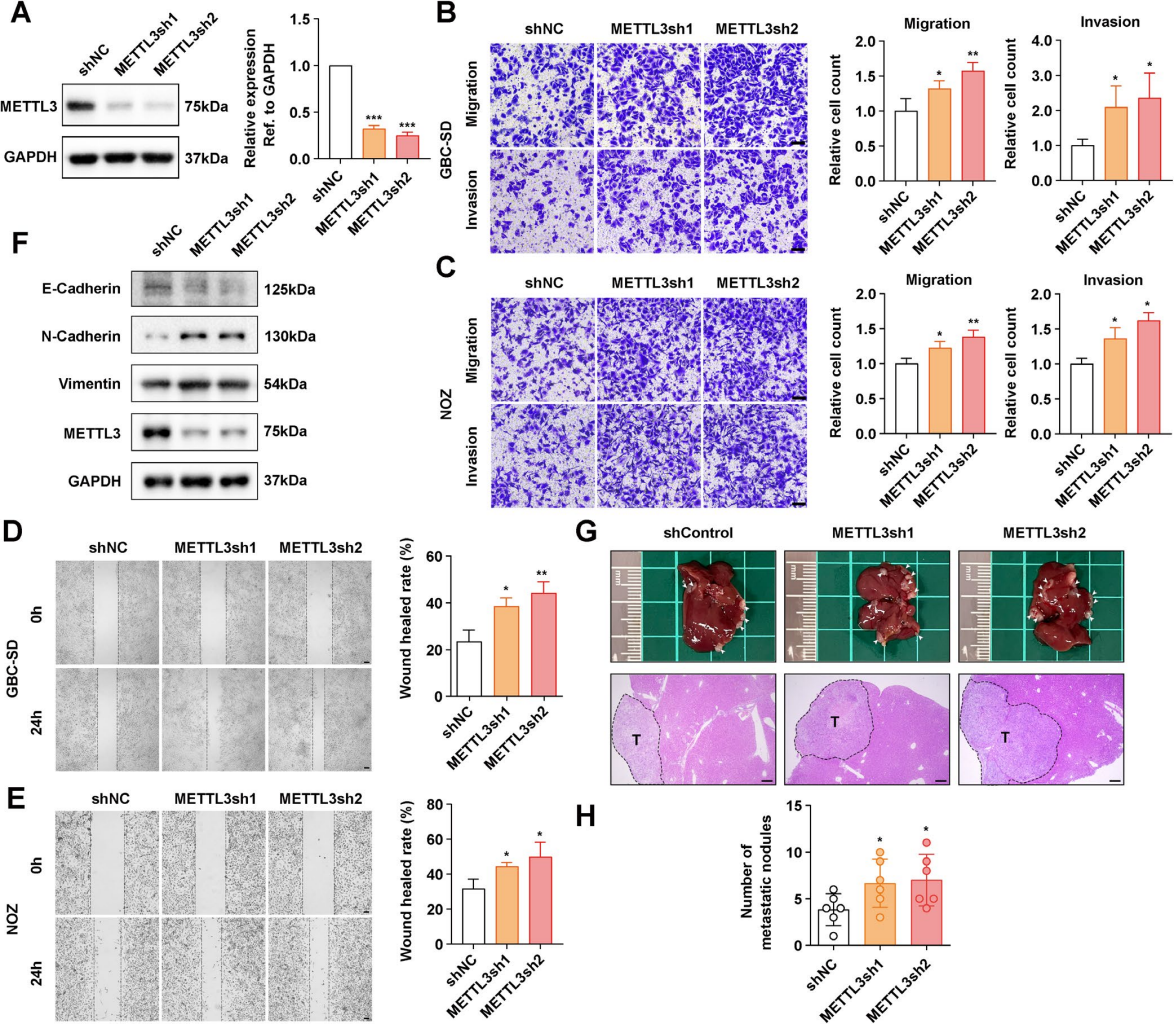
Reduced METTL3 enhanced the metastasis of GBC. (A) Western blot confirming knockdown of METTL3. (B, C) Transwell assay determining the migration and invasion ability of GBC cells with METTL3 knockdown. Scale bar: 100 μm. (D, E) Wound healing assay evaluating the migration ability of GBC cells with METTL3 knockdown. Scale bar: 100 μm. (F) Western blot assessing the influence of METTL3 knockdown on the expression of EMT‐related proteins. (G) Spleen‐liver metastasis model examining the metastasis ability of GBC cells in vivo. HE staining shows metastatic foci in the liver. T: tumour. Scale bar: 200 μm. (H) Statistical analysis of G. *p* values were calculated by Student's *t*‐test. *p < 0.05, **p < 0.01, ***p < 0.001.

Given the aberrant m6A levels and METTL3 and METTL14 expression levels, we proceeded to investigate their potential roles in the tumour biological behaviour of GBC. We established GBC cell lines with stable knockdown of METTL3 or METTL14 (Figures 2A, S1A). Transwell assays indicated that knockdown of either METTL3 or METTL14 markedly increased the invasiveness of GBC‐SD cells (Figures 2B,C, S1B). Consistent results were observed with a scratch wound healing assay (Figures 2D,E, S1C). Western blot analysis demonstrated that knockdown of METTL3 led to enhanced epithelial‐mesenchymal transition (EMT), as evidenced by decreased expression of E‐cadherin and increased expression of N‐cadherin and vimentin (Figure 2F). Subsequently, we evaluated the impact of METTL3 knockdown on the metastatic potential of GBC using a mouse spleen–liver metastasis model. The results revealed that METTL3 knockdown significantly increased the number of liver metastatic foci (Figure 2G,H). Taken together, these findings suggested that reduced m6A modification levels promote the invasive capacity of GBC cells.

### 3.3 Enhanced m6A Modification Suppresses Invasion and Metastasis in GBC

**FIGURE 3:**
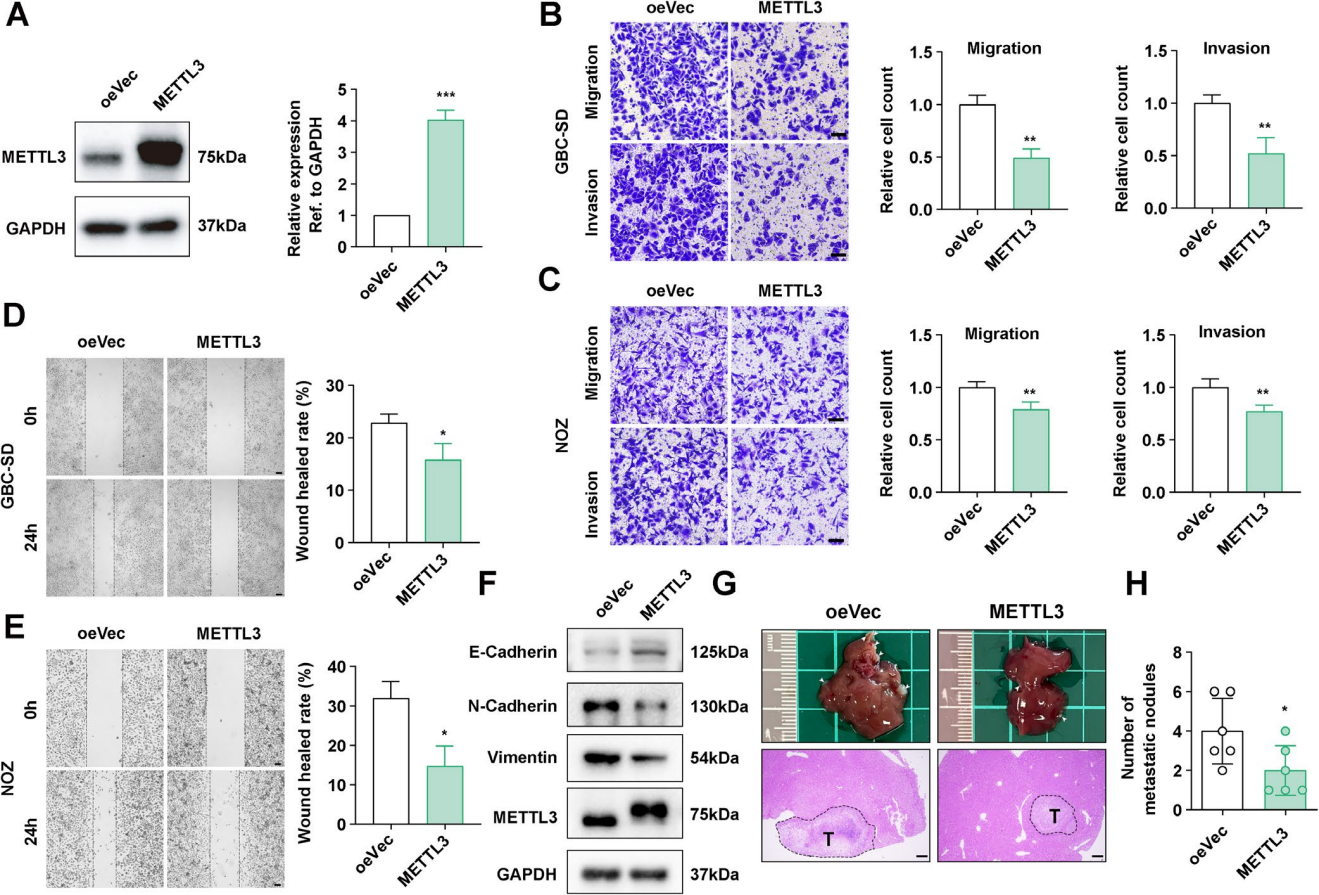
METTL3 overexpression restrained the metastasis of GBC. (A) Western blot confirming overexpression of METTL3. (B, C) Transwell assay determining the migration and invasion ability of GBC cells with METTL3 overexpression. Scale bar: 100 μm. (D, E) Wound healing assay evaluating the migration ability of GBC cells with METTL3 overexpression. Scale bar: 100 μm. (F) Western blot assessing the influence of METTL3 overexpression on the expression of EMT‐related proteins. (G) Spleen‐liver metastasis model examining the metastasis ability of GBC cells in vivo. HE staining shows metastatic foci in the liver. T: tumour. Scale bar: 200 μm. (H) Statistical analysis of G. *p* values were calculated by Student's *t*‐test. *p < 0.05, **p < 0.01, ***p < 0.001.

To further elucidate the influence of m6A modification levels on GBC invasion, we proceeded to establish GBC cells with stable overexpression of METTL3 or METTL14 (Figures 3A, S1D). Overexpression of either METTL3 or METTL14 significantly attenuated cell migration and invasion abilities, as evidenced by transwell assays (Figures 3B,C, S1E). Consistent results were obtained from the wound healing assay, which demonstrated decreased invasiveness in METTL3 or METTL14‐overexpressing GBC‐SD cells (Figures 3D,E, S1F). Western blot analysis further revealed that overexpression of METTL3 increased the expression of E‐cadherin while decreasing the expression of N‐cadherin and vimentin, suggesting that enhancement of m6A restricted EMT (Figure 3F). The mouse spleen–liver metastasis model corroborated these findings, showing that overexpression of METTL3 significantly suppressed liver metastasis in GBC (Figure 3G,H). Taken together, these results underscore the crucial roles of m6A modification and its core writers, METTL3 and METTL14, in regulating the invasion and metastasis of GBC.

### 3.4 M6A Inhibition Resulted in the Downregulation of miR‐146a‐5p in GBC

**FIGURE 4:**
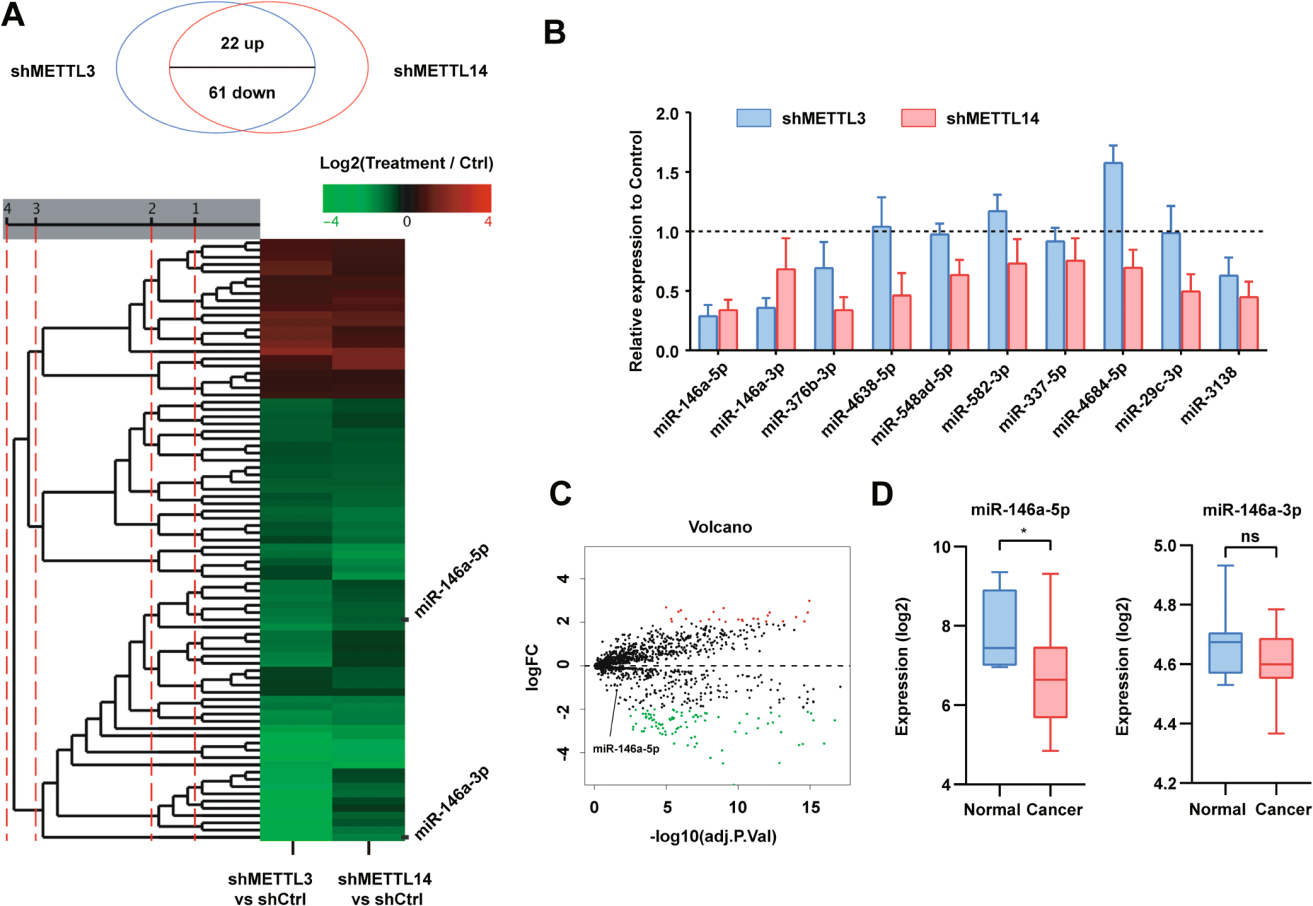
miR‐146a‐5p was downregulated in m^6^A‐reduced GBC cells. (A) Heatmap showing the alteration of microRNAs in METTL3 or METTL14 knockdown versus control GBC cells. (B) qRT‐PCR validation of indicated microRNAs in GBC cells. (C) Volcano plot showing the alteration of microRNAs in GBC tissues versus normal gallbladder tissues in GSE104165 datasets. (D) Analysis of miR‐146a‐5p and miR‐146a‐3p expression levels in GBC tissues versus normal gallbladder tissues in GSE104165 datasets. *p* values were calculated by Student's *t*‐test. *p < 0.05, ns, not significant.

Numerous miRNAs have been reported to regulate invasion and metastasis in gallbladder cancer [16, 20, 21]. Given the role of m6A in promoting the processing and maturation of miRNAs, coupled with the observed decrease in m6A modification levels in GBC, we hypothesised that these metastasis‐inhibiting miRNAs might be downregulated in GBC, potentially contributing to its highly invasive nature. This hypothesis prompted us to investigate alterations in miRNA levels in METTL3 and METTL14‐downregulated GBC cells through miRNA sequencing. Among the miRNAs that exhibited simultaneous changes in shMETTL3 and shMETTL14 GBC cells, 22 were upregulated while 61 were downregulated, with miR‐146a‐3p being the most significant downregulated miRNA. Additionally, miR‐146a‐5p showed significant downregulation in both shMETTL3 and shMETTL14 GBC cells (Figure 4A). Validation of downregulated miRNAs, including miR‐146a‐5p and miR‐146a‐3p, was performed by qRT‐PCR in shMETTL3 and shMETTL14 GBC cells, with miR‐146a‐5p emerging as the most markedly downregulated of the eight candidate miRNAs (Figure 4B). Further confirmation of our findings was obtained through the analysis of microarray data from the Gene Expression Omnibus database, which revealed differential expression of miRNAs in gallbladder cancer compared to non‐neoplastic gallbladder tissues (GSE104165) [22]. Volcano plots showed that among the differently expressed miRNAs, 26 were upregulated while 92 were downregulated in gallbladder cancer tissues compared to normal gallbladder tissues, aligning with our hypothesis (|LogFC| > 2, *p* < 0.05, Figures 4C, S2A). Analysis of miR‐146a‐5p and miR‐146a‐3p expression in this dataset revealed significantly lower levels of miR‐146a‐5p in gallbladder cancer tissues compared to normal gallbladder tissues, while miR‐146a‐3p did not exhibit significant differences (Figure 4D). Notably, miR‐146a‐5p emerged as the dominant miRNA among hsa‐miR‐146a. Thus, miR‐146a‐5p was selected for further investigation. Taken together, miR‐146a‐5p was selected for further investigation.

### 3.5 M6A Modification Promotes miR‐146a‐5p Maturation in a DGCR8‐Dependent Manner in GBC

**FIGURE 5:**
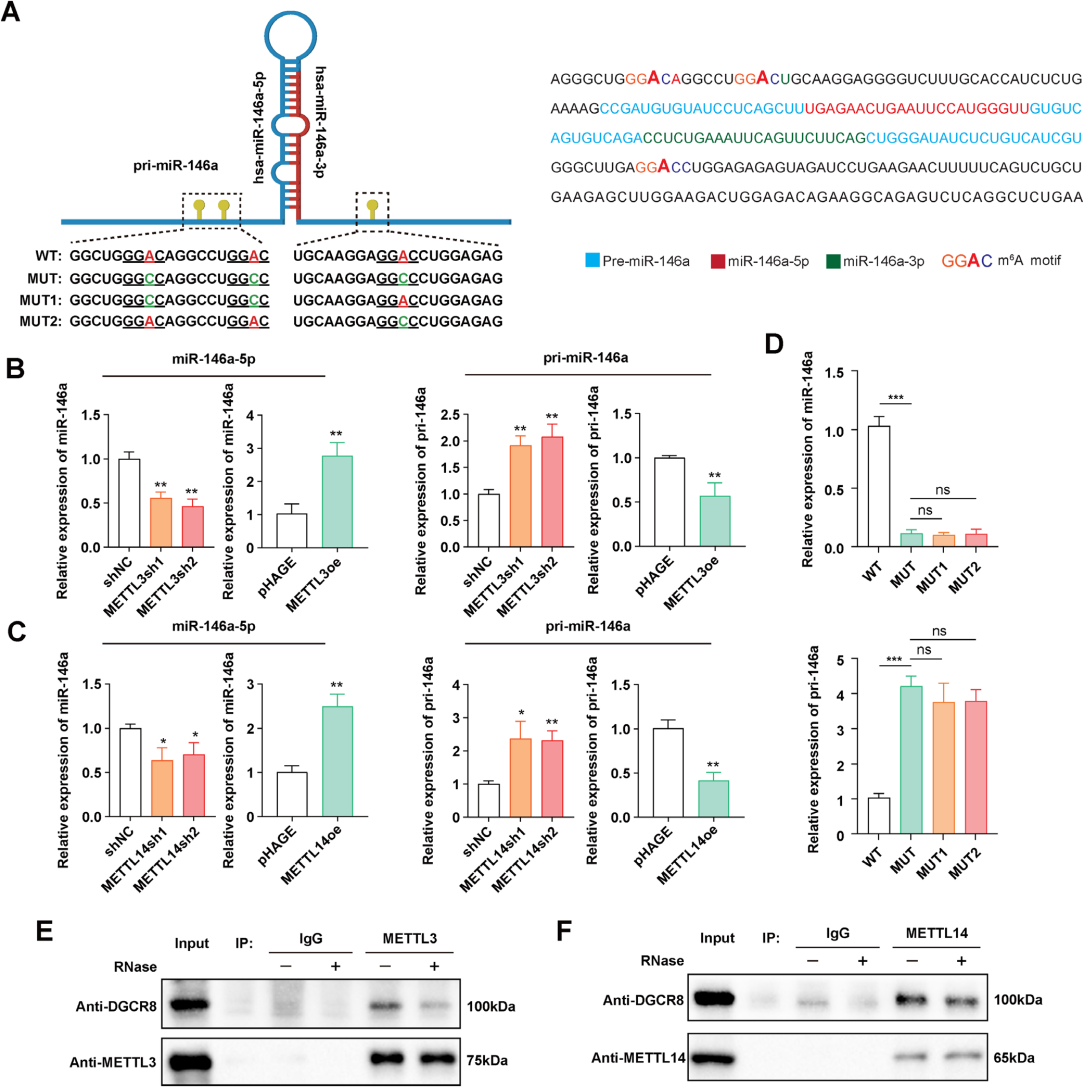
Maturation of miR‐146a‐5p was regulated in a m^6^A‐dependent manner. (A) Schematic showing the sequence of pri‐miR‐146a, pre‐miR‐146a, miR‐146a‐5p, miR‐146a‐3p, the potential m^6^A motif (GGAC) and its mutants in this study. (B, C) qRT‐PCR analysis of miR‐146a‐5p and pri‐miR‐146a expression levels in METTL3 or METTL14 knockdown or overexpression GBC cells. (D) Wild type or point mutant pri‐miR‐146a was transfected into GBC cells, and qRT‐PCR was performed to measure the expression levels of miR‐146a‐5p and pri‐miR‐146a. (E, F) Immunoprecipitation of METTL3 or METTL14 with DGCR8 with or without RNase treatment. *p* values were calculated by Student's *t*‐test. *p < 0.05, **p < 0.01, ns not significant.

Previous studies have underscored the pivotal role of m6A modification in promoting the processing of primary miRNAs [23, 24]. We proceeded to investigate whether METTL3 and METTL14 regulate the processing of pri‐miR‐146a in gallbladder cancer cells. The sequence of pri‐miR‐146a, pre‐miR‐146a, miR‐146a‐5p, and miR‐146a‐3p was characterised, and 3 potential m6A recognition sites (GGAC) were found within the pri‐miR‐146a sequence (Figure 5A). To ascertain whether METTL3 and METTL14 participated in the processing of pri‐miR‐146a into mature miR‐146a, we assessed the levels of pri‐miR‐146a and miR‐146a‐5p after depletion or overexpression of METTL3 and METTL14 in GBC cells using qRT‐PCR. Depletion of METTL3 or METTL14 led to the accumulation of pri‐miR‐146a and a reduction in mature miR‐146a‐5p levels. Conversely, METTL3 or METTL14 overexpression resulted in decreased pri‐miR‐146a levels and increased mature miR‐146a‐5p levels (Figure 5B,C), indicating that METTL3 and METTL14 promote the maturation of pri‐miR‐146a to miR‐146a‐5p. To investigate whether the maturation of miR‐146a‐5p depends on a specific m6A site in pri‐miR‐146a, we generated point mutations in pri‐miR‐146a, as shown in Figure 5A, and transfected the wildtype and mutated constructs into GBC cells. qPCR analysis revealed that mutation of any of the three GGAC motifs led to a significant reduction in miR‐146a‐5p levels, highlighting the critical role of m6A in the maturation of miR‐146a‐5p (Figure 5D). Next, we sought to determine whether aMETTL3 and METTL14 functioned by forming a complex with DGCR8. Immunoprecipitation assays proved that DGCR8 co‐precipitated with METTL3 and METTL14 in GBC cells (Figure 5E,F). Furthermore, this association was weakened under RNase treatment, suggesting the importance of RNA in their interactions. These findings indicate that METTL3 and METTL14 may facilitate pri‐miR‐146a processing by recruiting miRNA processor DGCR8 in GBC cells.

### 3.6 MiR‐146a‐5p Reverses the Migration and Invasion Ability of m6A‐Deficient GBC Cells

**FIGURE 6:**
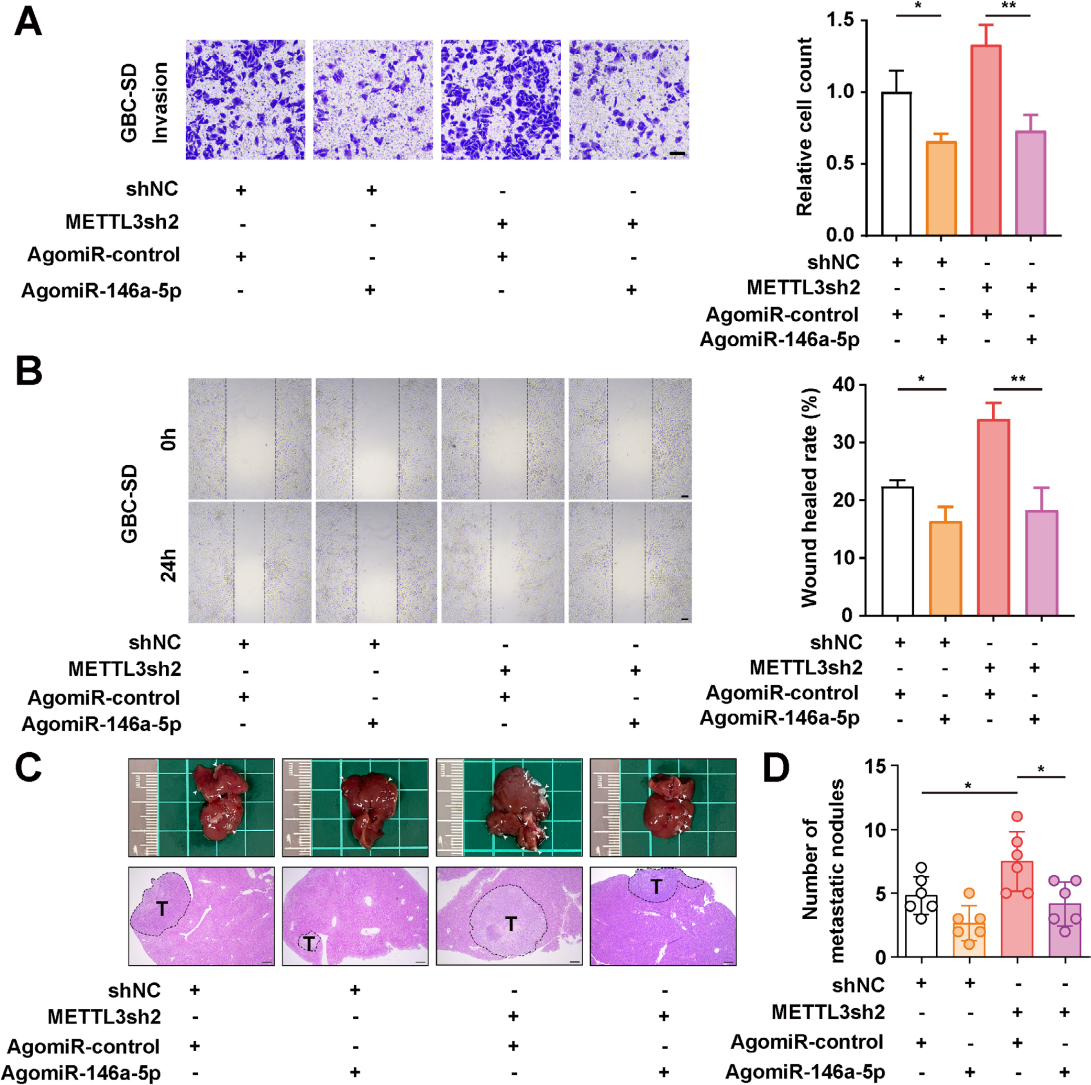
miR‐146a‐5p reversed the metastatic ability of m^6^A‐depleted GBC cells. (A) Transwell assay determining the migration and invasion ability of GBC cells with METTL3 knockdown and miR‐146a‐5p overexpression. Scale bar: 100 μm. (B) Wound healing assay evaluating the migration ability of GBC cells with METTL3 knockdown and miR‐146a‐5p overexpression. Scale bar: 100 μm. (C) Spleen‐liver metastasis model examining the metastasis ability of GBC cells in vivo. HE staining shows metastatic foci in the liver. T: tumour. Scale bar: 200 μm. (D) Statistical analysis of C. *p* values were calculated by Student's *t*‐test. *p < 0.05, **p < 0.01.

To determine whether the promotion of GBC cell invasiveness by m6A deletion is dependent on miR‐146a‐5p, we treated METTL3‐depleted GBC cells with agomiR‐146a. Wound healing assays and Matrigel transwell assays suggested that agomiR‐146a significantly suppressed the migration and invasion ability of METTL3‐depleted GBC cells (Figure 6A). Furthermore, the inhibitory effect of agomiR‐146a on the metastatic ability of GBC cells with METTL3 depletion was evaluated using a spleen‐liver metastasis model. As indicated in Figure 6B, agomiR‐146a‐5p treatment significantly reduced the number of liver metastatic nodules in the liver of the METTL3‐depleted group (Figure 6C,D). Taken together, our results indicated that m6A suppressed the migration and invasion abilities of GBC through miR‐146a‐5p.

## 4 Discussion

In our experiments, we found that m6A level, together with METTL3 and METTL14 expression levels, was decreased in GBC tissues compared to pair‐matched normal tissues. Functional experiments revealed that knockdown or overexpression of METTL3/METTL14 promoted or restrained the metastatic ability of GBC, respectively. Through miRNA‐sequencing and subsequent validation, we identified miR‐146a‐5p as a key downstream target regulated by m6A. Furthermore, our findings confirmed that miR‐146a‐5p maturation was dependent on m6A regulation of pri‐miR‐146a. Notably, agomiR‐146a‐5p reduced the invasiveness of m6A‐depleted GBC cells, underscoring its pivotal role in m6A deletion‐mediated GBC metastasis.

Increasing evidence has shed light on the association between dysregulated m6A levels and the development and progression of GBC. The m6A reader YTHDF2 has been implicated in promoting GBC progression and gemcitabine resistance by regulating DAPK3 in an m6A‐dependent manner [25]. Similarly, another m6A reader IGF2BP3 has been reported to promote GBC progression through various mechanisms, including activation of NF‐κB, YAP1/JUN, and AKT pathways [26, 27, 28]. These works primarily focused on m6A reader proteins which function by modulating the stability or translational efficiency of target mRNAs but did not evaluate the global m6A methylation levels or the expression or function of m6A writer enzymes. Recent studies also highlight the role of endogenous small‐molecule metabolites, such as acylcarnitines and deoxycholic acid, in regulating GBC progression via m6A‐dependent mechanisms. Accumulation of acylcarnitines was found to promote GBC migration by increasing m6A methylation on lncBCL2L11, thereby stabilising it and activating the THOC5/JNK axis to facilitate GBC metastasis [29]. On the other hand, deoxycholic acid inhibited GBC progression by interfering with METTL3 to impede m6A‐dependent miR‐92b‐3p maturation [30]. These studies focused on m6A changes in response to metabolite treatment; however, the overall m6A level in GBC compared to normal gallbladder tissues remained to be elucidated. Our study demonstrated that m6A levels, along with the expression levels of METTL3 and METTL14, were downregulated in GBC compared to normal gallbladder tissues, and decreased expression of METTL3 and METTL14 in GBC tissues were correlated with lymph node and distal organ metastasis and worse patient outcomes. This finding prompted us to investigate whether the diminished m6A level is associated with the aggressive biological behaviour of GBC. It is well‐established that m6A modification regulates key aspects of RNA metabolism, including pre‐mRNA processing, mRNA stability, translation efficiency, and miRNA biogenesis [9]. Among the effector proteins that regulate m6A biology, METTL3 and METTL14 form a core catalytic complex with the assistance of an additional subunit WTAP to function as adenosine methyltransferases responsible for the addition of m6A onto RNAs [8]. Therefore, we investigated the biological role of m6A in GBC by manipulating the expression of METTL3 and METTL14. Our results indicated that knockdown of METTL3 or METTL14 facilitated, while overexpression of METTL3 or METTL14 inhibited the migration and invasion ability of GBC cells, suggesting an association between m6A and the invasive potential of GBC. However, we did not interfere with the m6A‐demethylating enzymes FTO and ALKBH5, and the regulatory role of FTO or ALKBH5 in GBC has not been elucidated yet. Considering the complicated regulatory mechanism of m6A, future studies are warranted to verify these conclusions.

Recent studies have highlighted the crucial role of m6A in miRNA processing [31, 32]. It has been demonstrated that m6A was enriched in pri‐miRNAs and depletion of METTL3 leads to decreased m6A levels along with reduced generation of mature miRNAs [23]. They further identified HNRNPA2B1 as the reader of m6A that recognised m6A marks and recruited DGCR8. DGCR8, in turn, formed the microprocessor complex with the RNase III enzyme DROSHA to cleave pri‐miRNA into pre‐miRNA [23]. In the present study, we transfected GBC‐SD cells with shRNAs targeting METTL3 or METTL14 and investigated the alterations of downstream miRNAs by miRNA sequencing. We observed simultaneous downregulation of miR‐146a‐3p and miR‐146a‐5p in shMETTL3 and shMETTL14 GBC cells, which was further confirmed by qRT‐PCR. Consistent with our hypothesis, analysis of GEO datasets GSE104165 submitted by Goeppert et al. indicated a predominance of significantly downregulated miRNAs compared to upregulated miRNAs, with lower levels of miR‐146a‐5p observed in gallbladder cancer tissues compared to normal gallbladder tissues. Moreover, we identified three GGAC motifs in pri‐miR‐146a, which might be recognised by the methyltransferase complex. Mutation of any of the three GGAC motifs led to a significant reduction in miR‐146a‐5p levels, highlighting the critical role of m6A in the maturation of miR‐146a‐5p. Depletion or forced expression of METTL3 and METTL14 inhibited or promoted the processing of pri‐miR‐146a, respectively. Co‐IP assay proved that METTL3 and METTL14 interacted with DGCR8 in an RNA‐dependent manner in GBC cells. Taken together, our results indicated that METTL3 and METTL14 promoted the processing of pri‐miR‐146a in an m6A‐dependent manner in GBC cells.

Human miR‐146a‐5p, reported by Taganov et al. in 2006, was initially implicated in inflammation and immune regulation by downregulating the NF‐κB p65 signalling pathway [33]. Subsequent studies have increasingly focused on its role in the development and progression of various cancer types. Contreras et al. demonstrated that miR‐146a‐5p inhibited the transcription factor EGR1 and its downstream genes, thereby suppressing the growth of B‐cell lymphoma [34]. Kumaraswamy et al. identified miR‐146a‐5p as a tumour suppressor in breast cancers, where BRCA1 facilitates its transcription, leading to inhibition of EGFR [35]. Additionally, Karthikeyan et al. elucidated the role of miR‐146a‐5p in glioma by targeting SMAD4 to suppress the tumorigenic gene MM9, thereby impeding glioma growth and progression [36]. Specifically, miR‐146a‐5p has been reported to bind directly to WNT2 [37], EGFR [38], NOTCH2 [39], TRAF6 [40], IRAK1 [41] and EIF5A2 [42] to inhibit EMT and act as a tumour suppressor in various human cancers. In our study, we found that miR‐146a‐5p inhibited the migration and invasion abilities of gallbladder cancer cells both in vitro and in vivo. Furthermore, agomiR‐146a‐5p effectively suppressed the migration and invasion abilities of m6A‐depleted GBC cells both in vitro and in vivo. Collectively, our findings suggest that miR‐146a‐5p acted as a tumour suppressor and was regulated in a m6A‐dependent manner in GBC. Given that METTL3 and METTL14 expression is reduced in GBC and contributes to decreased miR‐146a‐5p levels and enhanced metastasis, therapeutic strategies aimed at restoring miR‐146a‐5p expression may offer clinical benefit. For instance, liposomal or nanoparticle‐based delivery of synthetic miR‐146a‐5p mimics represents a promising approach to restore its tumour‐suppressive activity. In addition, as gemcitabine remains a cornerstone chemotherapeutic agent in GBC, combination regimens involving miR‐146a‐5p replacement and gemcitabine chemotherapy may offer synergistic effects by simultaneously suppressing EMT and inhibiting proliferation.

## 5 Conclusions

**FIGURE 7:**
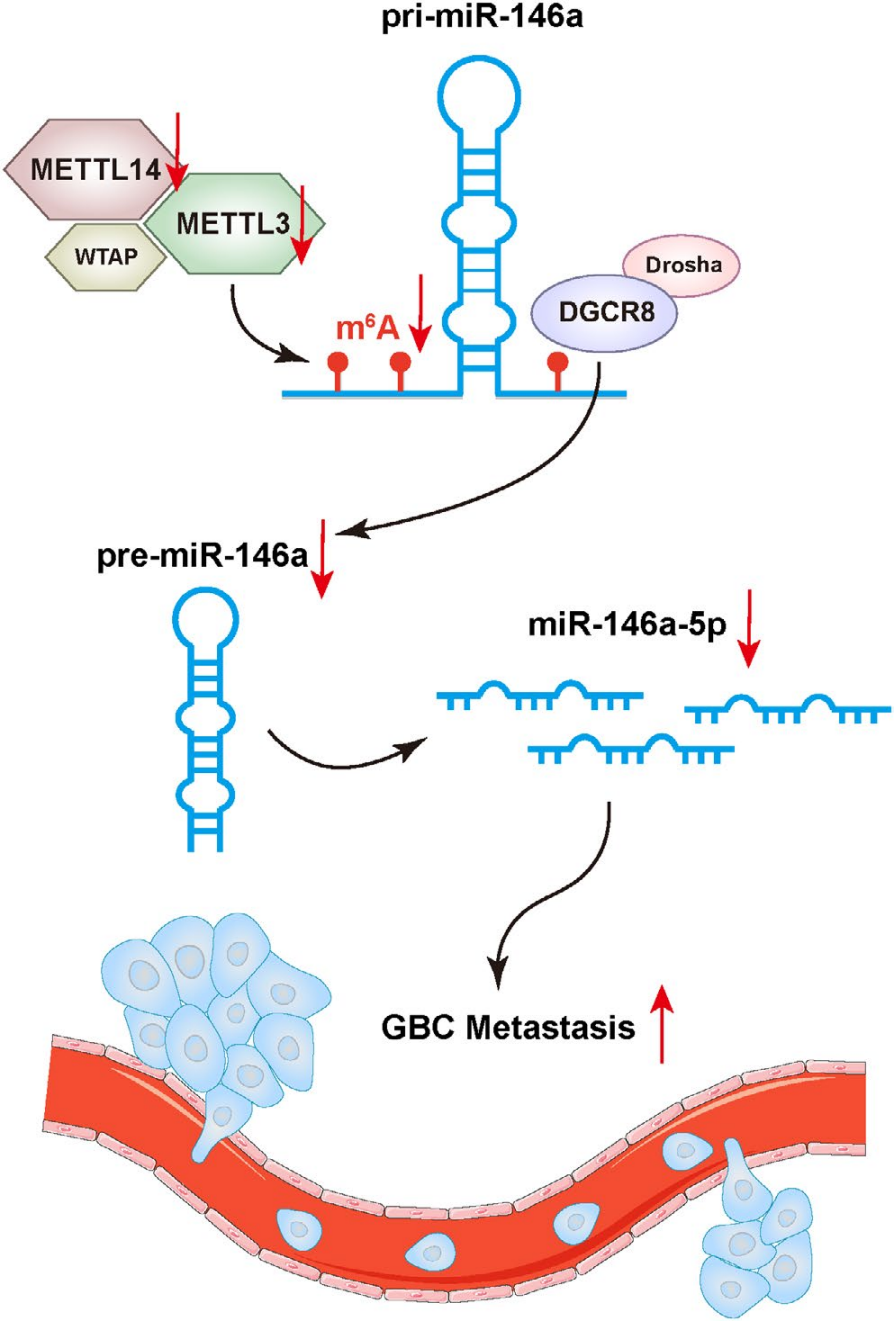
Schematic representation of the study.

Our study provides compelling evidence that the m6A modification, as well as the core methyltransferase units, METTL3 and METTL14, is downregulated in GBC. This downregulation leads to decreased expression of miR‐146a‐5p, which in turn promotes the migration and invasion abilities of GBC cells both in vitro and in vivo (Figure 7). Our study further supported the m6A‐mediated miRNA processing hypothesis in GBC cells and underscored the critical role of m6A‐regulated miRNA maturation in the pathogenesis of gallbladder cancer. Targeting the m6A‐miRNA regulatory machinery may hold promise for the development of novel therapeutic strategies for GBC treatment.

## Author Contributions

**Yuhui Liu:** conceptualization (equal), data curation (equal), formal analysis (equal), funding acquisition (equal), investigation (equal), visualization (equal), writing – original draft (equal), writing – review and editing (equal). **Ruizhi He:** conceptualization (equal), data curation (equal), funding acquisition (equal), investigation (equal), validation (equal), visualization (equal), writing – original draft (equal). **Wenjia Wang:** data curation (equal), investigation (equal), methodology (equal), validation (equal), visualization (supporting), writing – original draft (supporting). **Qilong Xia:** investigation (equal), methodology (equal), software (equal), validation (equal), visualization (equal). **Di Zhang:** investigation (equal), methodology (equal), software (equal), validation (equal), visualization (equal). **Shutao Pan:** data curation (equal), funding acquisition (equal), methodology (equal). **Min Wang:** methodology (equal), supervision (equal), writing – review and editing (equal). **Qi Zhang:** methodology (equal), writing – review and editing (equal). **Simiao Xu:** conceptualization (equal), project administration (equal), supervision (equal), writing – review and editing (equal). **Jun Gong:** conceptualization (equal), investigation (equal), project administration (equal), supervision (equal), writing – review and editing (equal). **Renyi Qin:** conceptualization (lead), funding acquisition (equal), project administration (lead), supervision (lead), writing – review and editing (equal).

## Ethics Statement

The study was conducted in accordance with the Declaration of Helsinki and approved by the Human Research Ethics Committee of Tongji Hospital of Huazhong University of Science and Technology. Animal studies were approved by the Laboratory Animal Welfare and Ethics Committee of Tongji Hospital, Tongji Medical College, Huazhong University of Science and Technology.

## Conflicts of Interest

The authors declare no conflicts of interest.

## Supporting information


**Data S1:** jcmm70764‐sup‐0001‐Supinfo.docx.

## Data Availability

All data are available from the corresponding author upon reasonable request.
