# Supplementary material for: Dysregulated RNA m6A Methylation Contributes to the Metastasis of Gallbladder Cancer Through miR‐146a‐5p
Source: J Cell Mol Med. 2025 Aug 10;29(15):e70764. doi: 10.1111/jcmm.70764 (PMC12335937; doi:10.1111/jcmm.70764)
Supplement: Supplementary file 1 — Data S1: jcmm70764‐sup‐0001‐Supinfo.docx. [file JCMM-29-e70764-s001.docx]

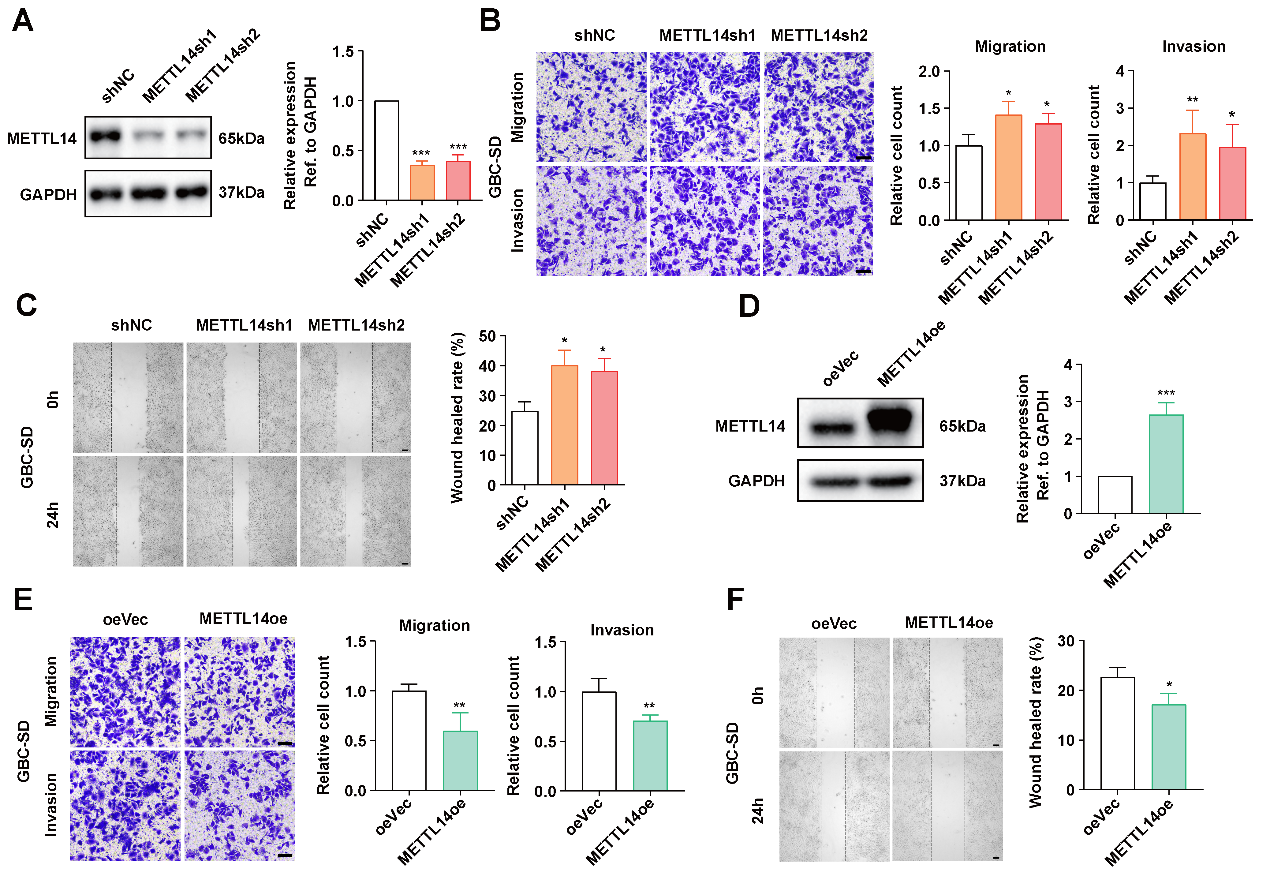


**Fig. S1** Influence of METTL14 on GBC metastasis. **A** Western blot confirming knockdown of METTL14. **B-C** Transwell assay determining the migration and invasion ability of GBC cells with METTL14 knockdown. Scale bar: 100 μm. **D** Western blot confirming overexpression of METTL14. **E-F** Transwell assay evaluating the migration and invasion ability of GBC cells with METTL14 overexpression. Scale bar: 100 μm. *P* values were calculated by Student’s *t*-test. **P* < 0.05, ***P* < 0.01, ****P* < 0.001.

**
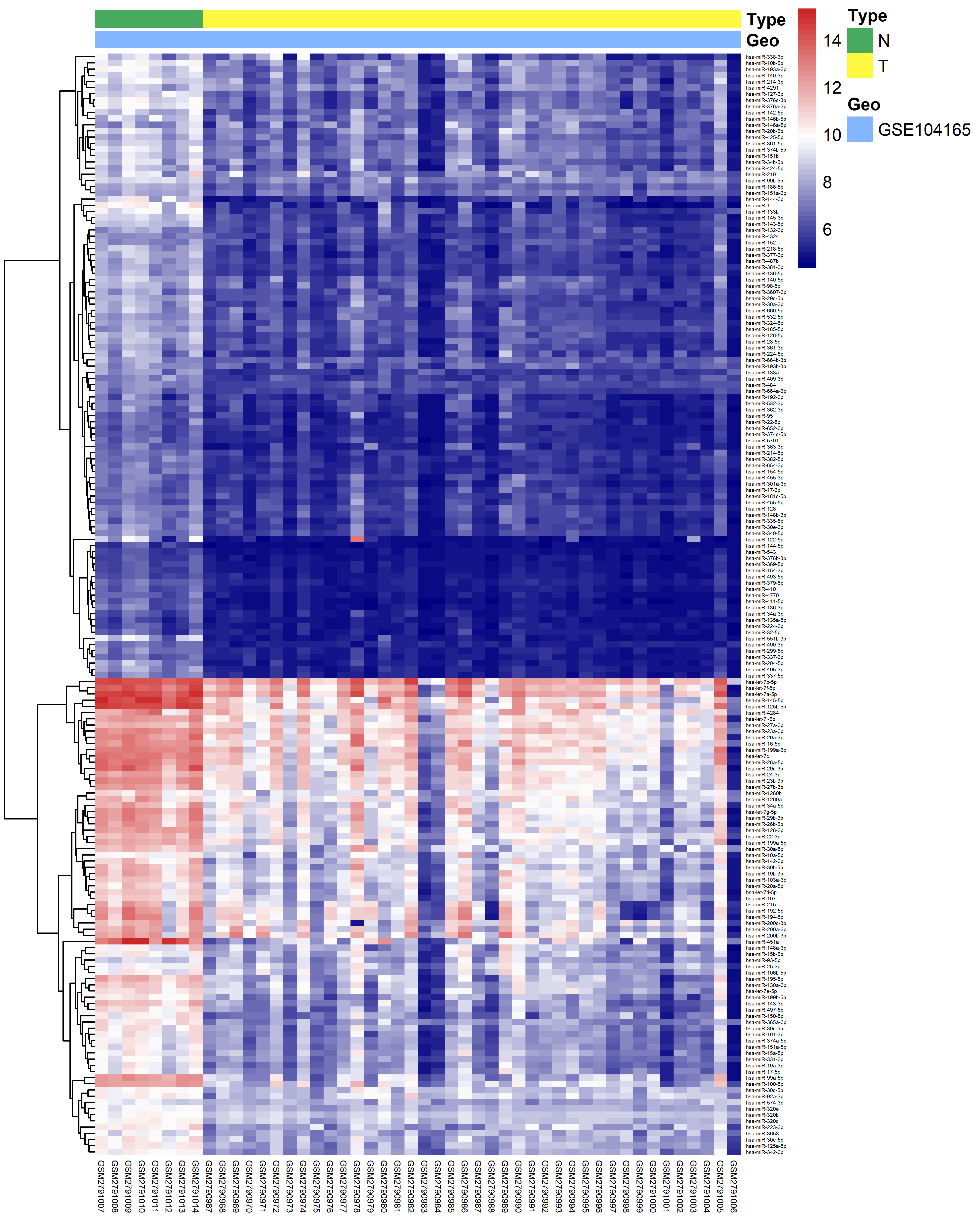
**

**Fig. S2** Expression of microRNAs in GBC and normal gallbladder tissues (GSE104165). Heatmap showing expression of microRNAs in GBC and normal gallbladder tissues in GSE104165 datasets.

**Supplementary Table 1. Sequence of shRNA, qPCR primers for mRNA, microRNA used in this study.**

Stem-loop qPCR primers

| miRNAs | Stem-loop primers |
| --- | --- |
| miR-146a-5p | GTCGTATCCAGTGCAGGGTCCGAGGTATTCGCACTGG  ATACGACAACCCA |
| miR-146a-3p | GTCGTATCCAGTGCAGGGTCCGAGGTATTCGCACTGG  ATACGACCTGAAG |
| miR-376b-3p | GTCGTATCCAGTGCAGGGTCCGAGGTATTCGCACTGG  ATACGACAACATG |
| miR-4638-5p | GTCGTATCCAGTGCAGGGTCCGAGGTATTCGCACTGG  ATACGACACTTGT |
| miR-548ad-5p | GTCGTATCCAGTGCAGGGTCCGAGGTATTCGCACTGG  ATACGACCAAAAA |
| miR-582-3p | GTCGTATCCAGTGCAGGGTCCGAGGTATTCGCACTGG  ATACGACGGTTCA |
| miR-337-5p | GTCGTATCCAGTGCAGGGTCCGAGGTATTCGCACTGG  ATACGACAACTCC |
| miR-4684-5p | GTCGTATCCAGTGCAGGGTCCGAGGTATTCGCACTGG  ATACGACTATGTT |
| miR-29c-3p | GTCGTATCCAGTGCAGGGTCCGAGGTATTCGCACTGG  ATACGACTAACCG |
| miR-3138 | GTCGTATCCAGTGCAGGGTCCGAGGTATTCGCACTGG  ATACGACACTCCC |

qPCR primers for miRNAs

| miRNA | Forward | Reverse |
| --- | --- | --- |
| miR-146a-5p | CGCGTGAGAACTGAATTCCA | AGTGCAGGGTCCGAGGTATT |
| miR-146a-3p | CGCGCCTCTGAAATTCAGTT | AGTGCAGGGTCCGAGGTATT |
| miR-376b-3p | GCGCGATCATAGAGGAAAATC | AGTGCAGGGTCCGAGGTATT |
| miR-4638-5p | GACTCGGCTGCGGTGG | AGTGCAGGGTCCGAGGTATT |
| miR-548ad-5p | CGCGCGAAAAGTAATTGTGG | AGTGCAGGGTCCGAGGTATT |
| miR-582-3p | GCGCGTAACTGGTTGAACAAC | AGTGCAGGGTCCGAGGTATT |
| miR-337-5p | CGCGGAACGGCTTCATACA | AGTGCAGGGTCCGAGGTATT |
| miR-4684-5p | CGCGCTCTCTACTGACTTGC | AGTGCAGGGTCCGAGGTATT |
| miR-29c-3p | CGCGTAGCACCATTTGAAAT | AGTGCAGGGTCCGAGGTATT |
| miR-3138 | GCGTGTGGACAGTGAGGTAGA | AGTGCAGGGTCCGAGGTATT |
| U6 | CTCGCTTCGGCAGCACA | AACGCTTCACGAATTTGCGT |

qPCR primers for mRNAs or primary-miRNAs

| Gene | Forward | Reverse |
| --- | --- | --- |
| GAPDH | GGAGCGAGATCCCTCCAAAAT | GGCTGTTGTCATACTTCTCATGG |
| Pri-miR-146a | ACAGGCCTGGACTGCAAGGA | CAGGATCTACTCTCTCCAGGTCCTC |
| Puromycin | ATGACCGAGTACAAGCCCAC | ACACCTTGCCGATGTCGAG |

The target sequence for shRNAs

| Target gene | Target Sequence |
| --- | --- |
| shMETTL3#1 | GCAAGTATGTTCACTATGAAA |
| shMETTL3#2 | GCTGCACTTCAGACGAATTAT |
| shMETTL14#1 | GAACCTGAAATTGGCAATATA |
| shMETTL14#2 | GCCGTGGACGAGAAAGAAATA |
